# Metformin Attenuates Inflammation and Fibrosis in Thyroid-Associated Ophthalmopathy

**DOI:** 10.3390/ijms232415508

**Published:** 2022-12-07

**Authors:** Zhihui Xu, Huijing Ye, Wei Xiao, Anqi Sun, Shenglan Yang, Te Zhang, Xiaotong Sha, Huasheng Yang

**Affiliations:** State Key Laboratory of Ophthalmology, Zhongshan Ophthalmic Center, Sun Yat-sen University, Guangdong Provincial Key Laboratory of Ophthalmology and Visual Science, Guangzhou 510060, China

**Keywords:** thyroid-associated ophthalmopathy, metformin, AMPK, orbital fibroblast

## Abstract

The pathogenesis of thyroid-associated ophthalmopathy (TAO) is still unclear, and therapeutic drugs have great limitations. As metformin has multiple therapeutic effects in many autoimmune diseases, we explored the effects of metformin on TAO in an in vitro fibroblast model. We used orbital connective tissues and fibroblasts that were obtained from TAO patients and normal controls. The activity of adenosine monophosphate-activated protein kinase (AMPK) and the levels of inflammatory or fibrotic factors were examined by immunofluorescence (IF) and immunohistochemistry (IHC). Quantitative real-time polymerase chain reaction (qPCR), cytokine quantification by enzyme-linked immunosorbent sssay (ELISA), IF, and western blotting (WB) were used to measure the expression of factors related to inflammation, fibrosis, and autophagy. To determine the anti-inflammatory and antifibrotic mechanisms of metformin, we pretreated cells with metformin, 5-aminoimidazole-4-carboxamide 1-β-D-ribofuranoside (AICAR, an AMPK activator) or compound C (CC, an AMPK inhibitor) for 24 h and used WB to verify the changes in protein levels in the AMPK/mammalian target of rapamycin (mTOR) pathway. We determined that the low activity of AMPK in the periorbital tissue of TAO patients may be closely related to the occurrence and development of inflammation and fibrosis, and metformin exerts multiple effects by activating AMPK in TAO. Furthermore, we suggest that AMPK may be a potential target of TAO therapy.

## 1. Introduction

Thyroid-associated ophthalmopathy (TAO) is an organ-specific autoimmune disease characterized by invasive lesions in the retrobulbar and periorbital ocular tissues that is also known as Grave’s ophthalmopathy. Approximately 3 to 5% of patients may experience severe symptoms such as eye pain, vision-threatening corneal ulcers, or compressive optic neuropathy [1,2,3,4]. TAO is pathologically characterized by orbital inflammation, hyaluronic acid synthesis, fibrosis, and tissue remodeling. Early lesions cause inflammation, congestion, and tissue expansion, which manifest as eye pain, conjunctiva and eyelid swelling, proptosis, and even exposure keratopathy and compressive optic neuropathy. The next phase is chronic fibrosis with irreversible localized strabismus. TAO greatly reduces the quality of life [5].

Currently, TAO is commonly treated with glucocorticoids, irradiation or surgery, but the long-term use of glucocorticoids can cause serious side effects in patients, including Cushing’s syndrome, diabetes, and hypertension [6]. In addition, some patients do not respond well to glucocorticoid therapy [7,8,9]. Novel targeted drugs have gradually been developed, and these include teprotumumab (anti-IGF-1R monoclonal antibody) [10,11] and rituximab (anti-CD20 monoclonal antibody) [12,13]. These new drugs have ahigh therapeutic efficiency, but the biological agents and targeted medicines are generally very expensive; moreover, research is needed on the effectiveness, safety, and optimal doses of drugs for TAO. Therefore, the development of therapeutic drugs is a hotspot of current TAO research.

The pathogenesis of TAO remains unclear. It is currently believed that the occurrence of TAO depends on the effect of TSHR autoantibodies and TSHR-specific T cells on TSHR expressed in nonthyroid tissues, particularly fibroblasts and adipocytes [14]. Immune cells such as T and B cells are activated during the disease process of TAO [15], and our previous research confirmed this finding [16]. These immune cells infiltrate the periorbital connective tissue and lead to the production of cytokines such as IL-1β, IL-6, IL-8, TNF-α, RANTES, and TGF-β1 and autoantibodies such as IgG [17,18,19], which ultimately leads to inflammation in connective tissue and tissue fibrosis and remodeling.

Adenosine monophosphate-activated protein kinase (AMPK) is a key cellular bioenergy sensor and metabolic regulator [20,21]. AMPK activation leads to the inactivation of energy consumption pathways and the activation of energy production pathways and thereby restores energy homeostasis [22]. Studies have shown that decreased AMPK activity is associated with diabetes, obesity, aging [23,24,25], tissue inflammation, and fibrosis [26,27,28], particularly in adipose tissue [29,30]. In addition, AMPK activation can inhibit myofibroblast differentiation through TGF-β1, prevent the development of fibrosis [31,32], or even reverse established fibrosis [33]. Moreover, mammalian target of rapamycin (mTOR) and AMPK activate autophagy in response to low energy levels [34]. In conclusion, we hypothesize that the local tissue of TAO patients has decreased AMPK activity, which is closely related to the occurrence of inflammation and fibrosis.

Metformin, an AMPK activator that is widely used in the treatment of diabetes and activates catabolic pathways through the phosphorylation of AMPK [35] exerts hypoglycemic and weight loss effects [36]. To date, metformin has been used in animal models and in vitro cell models of uveitis [37], systemic lupus erythematosus [38], infection diseases [39], inflammatory diseases [40], and idiopathic pulmonary fibrosis [33,41]. We hypothesize that metformin plays an inhibitory role in disease processes that are characteristic of TAO, and we aimed to determine the therapeutic effects of metformin on these parameters and the signaling pathways involved.

## 2. Results

### 2.1. Decreased AMPK Activity in the Orbital Connective Tissue of TAO Patients

**Figure 1:**
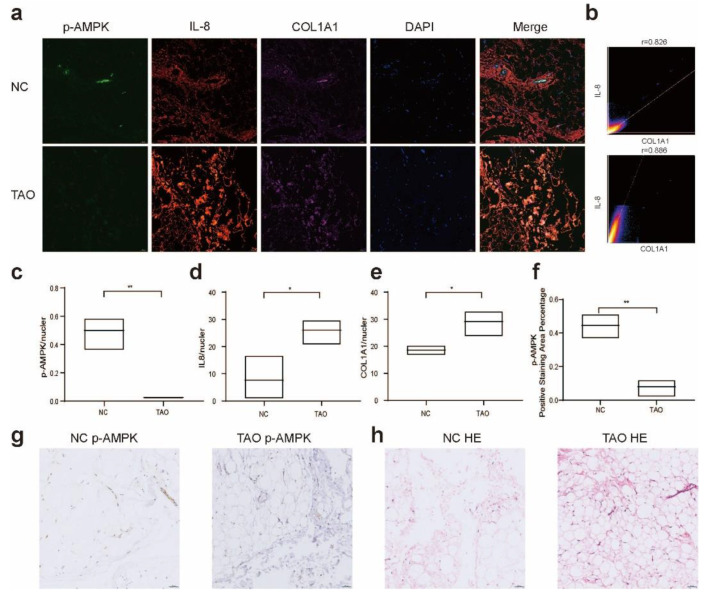
Alterations in AMPK activity in TAO orbital connective tissue. (**a**) Four-color IF analysis showing IL-8 (red), p-AMPK (green), COL1A1 (purple), and nuclei (blue) in the connective tissue of TAO and NC samples. Colocalization of p-AMPK, inflammation and fibrosis markers in orbital tissues of different populations. Scale bar, 50 µm. (**b**) Scattergrams indicate the fluorescence intensity and Pearson’s correlation (r) of IL-8 and COL1A1 in the image in (**a**). (**c**–**e**) Ratio of the fluorescence intensity of p-AMPK, IL-8, and COL1A1 to nuclei. (**f**) Average optical density (AOD) of p-AMPK in each group shown in (**g**). (**g**,**h**) DAB staining of p-AMPK and HE staining of tissue in the TAO and NC groups. Scale bar, 50 µm. *n* = 3 (control), *n* = 3 (TAO) for all. * *p* < 0.05, ** *p* < 0.01 (unpaired *t* test).

The orbital connective tissue of TAO patients was characterized by the presence of inflammatory molecules, such as IL-8, in the fibroblast foci, and the fibroblasts expressed high levels of collagen, resulting in structural tissue remodeling (Figure 1a,b,d,e). Importantly, within these areas of active inflammation and fibrosis, we observed a significant reduction in AMPK activity (Figure 1a,c,f,g). This finding indicates lower AMPK activity in the periorbital tissue of TAO patients, which may be related to the high levels of inflammation and fibrosis in the tissue. Thus, we hypothesized that the activation of AMPK could regulate the metabolism of TAO fibroblasts and reduce the levels of inflammatory and fibrotic proteins and thus exert a certain therapeutic effect.

### 2.2. Fibroblast Identification and Effects of Different Concentrations of Metformin

**Figure 2:**
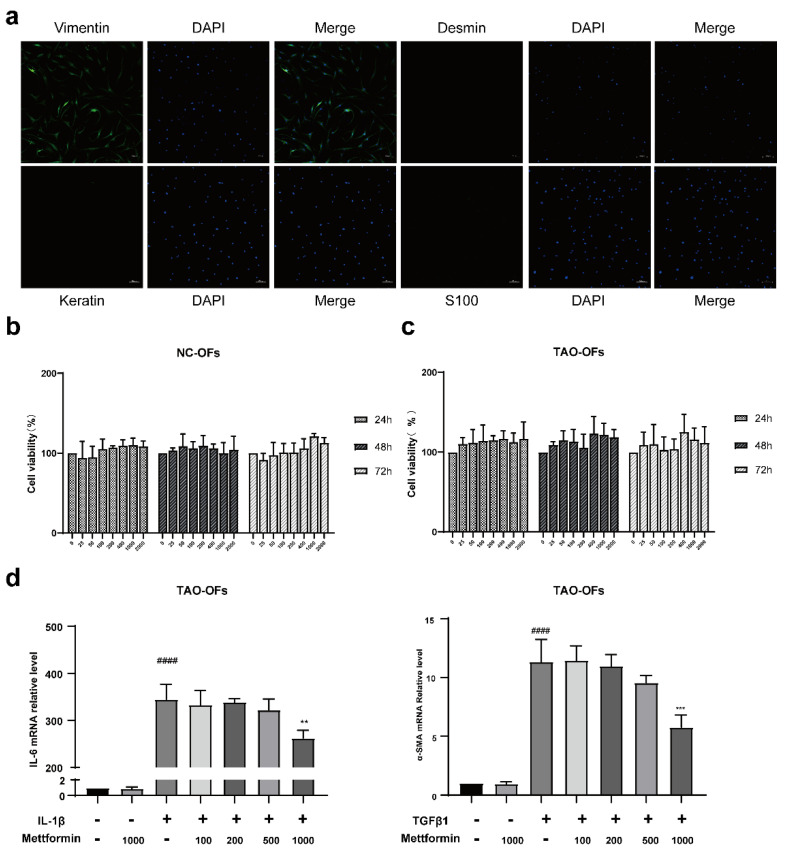
Fibroblast identification and determination of metformin in vitro. (**a**) IF analysis of the expressions of vimentin, desmin, keratin, and s100. Scale bar, 100 µm. (**b**,**c**) CCK8 assay results showing that the different concentrations of metformin did not affect the viability of TAO and NC OFs. (**d**) The mRNA expressions of IL-6 and α-SMA in TAO-OFs treated with different concentrations of metformin were determined by qPCR. #### *p* < 0.0001, compared with the control group, ** *p* < 0.01, *** *p* < 0.001 compared with the IL-1β or TGF-β1 group (ANOVA).

IF analysis showed positive staining for only vimentin and negative staining for other proteins. This finding proved that the primary and passaged cells used in the experiment were fibroblasts (Figure 2a). The CCK8 results revealed no significant difference in cell numbers between the control and metformin (Figure 2b,c). Moreover, we tested the effect of different concentrations of metformin on the mRNA levels of the inflammatory factor IL-6 and the fibrotic factor SMA and found that only 1000 µM metformin significantly reduced the levels of these factors (Figure 2d). Thus, we chose 1000 µM metformin for the subsequent experiments.

### 2.3. Metformin Inhibits the Expression of Inflammatory Molecules

**Figure 3:**
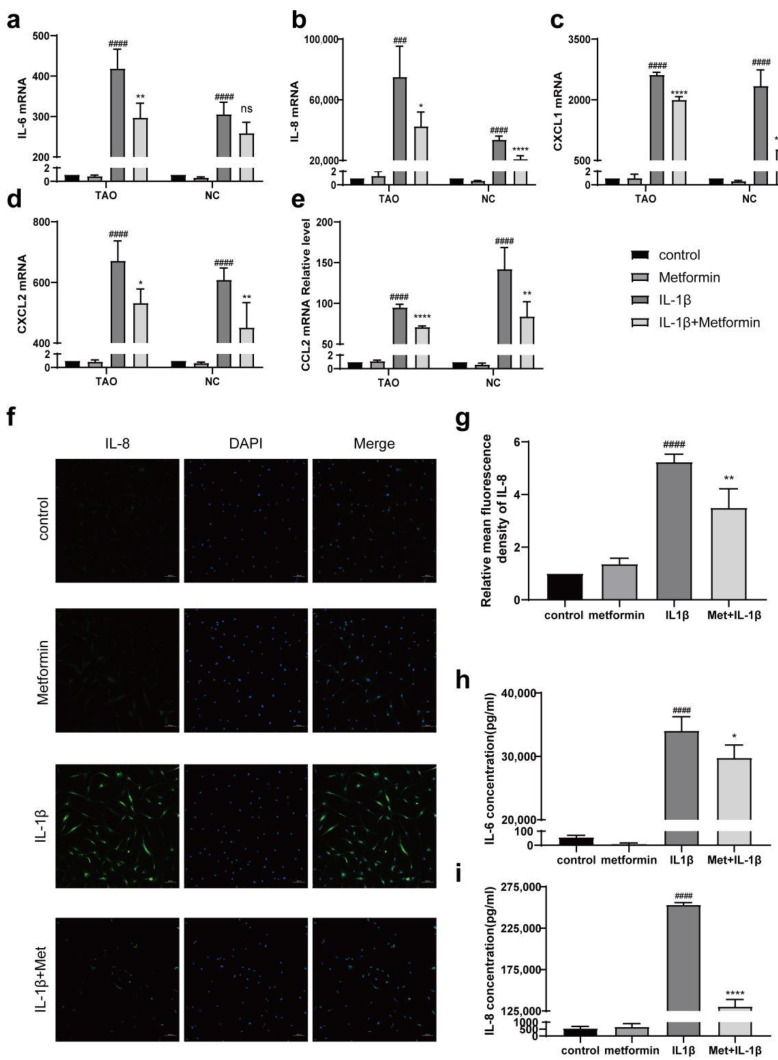
Effects of metformin on IL1β-induced inflammation. (**a**–**e**) TAO-OFs and NC-OFs were pretreated with metformin for 24 h and then stimulated with 1 ng/mL IL-1β for 24 h. The mRNA expression of proinflammatory cytokines and chemokines was determined by qPCR. (**f**) IF analysis of the expression of IL-8. Scale bar, 100 µm. The quantitative analysis is shown in (**g**). (**h**,**i**) The expressions of IL-6 and IL-8 in cell cultures. *n* = 3 in each group. ### *p* < 0.001, #### *p* < 0.0001 compared with the control group, * *p* < 0.05, ** *p* < 0.01, **** *p* < 0.0001 compared with the IL-1β group, ns: not significant (ANOVA).

We cultured TAO OFs with IL-1β (1 ng/mL) for 24 h. qPCR confirmed that metformin significantly inhibited the IL-1β-induced mRNA expression of proinflammatory cytokines and chemokines, including IL-6, IL-8, CXCL1, CXCL2, and CCL2 (Figure 3a–e). Compared with NC OFs, TAO-OFs more notably inhibited inflammation at most time points. In addition, metformin inhibited the protein expression of IL-8 (Figure 3f,g). We found that the cells treated with both metformin and IL-1β had lower levels of IL-6 and IL-8 than the cells treated with IL-1β without metformin (Figure 3h,i).

### 2.4. Metformin Inhibits HA Production

**Figure 4:**
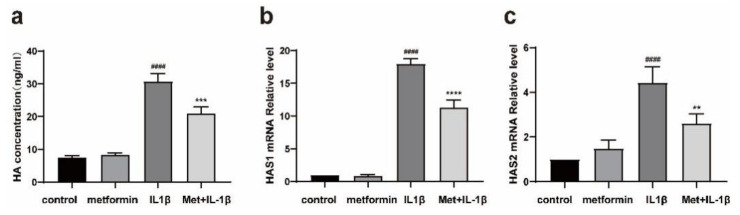
Metformin reduces HA production. (**a**) TAO-OFs were pretreated with metformin for 24 h and then stimulated with 1 ng/mL IL-1β for 24 h. The ELISA results show the changes in the HA levels after stimulation and treatment. (**b**,**c**) Quantitative analysis of HAS1 and HAS2 mRNA levels. *n* = 3 in each group. #### *p* ≤ 0.0001, compared with the control group, ** *p* ≤ 0.01, *** *p* ≤ 0.001, **** *p* ≤ 0.0001, compared with the IL-1β group. (ANOVA).

ELISA confirmed that metformin inhibited HA production in the OF inflammation model. IL-1β increased the HA levels in TAO OFs compared with those in untreated control cells, whereas pretreatment with metformin suppressed HA production (Figure 4a). Furthermore, HAS1 and HAS2 mRNA expressions were significantly upregulated in the stimulation group compared with the control group, and these indices were significantly decreased after pretreatment with metformin (Figure 4b,c).

### 2.5. Metformin Inhibits Fibrosis

**Figure 5:**
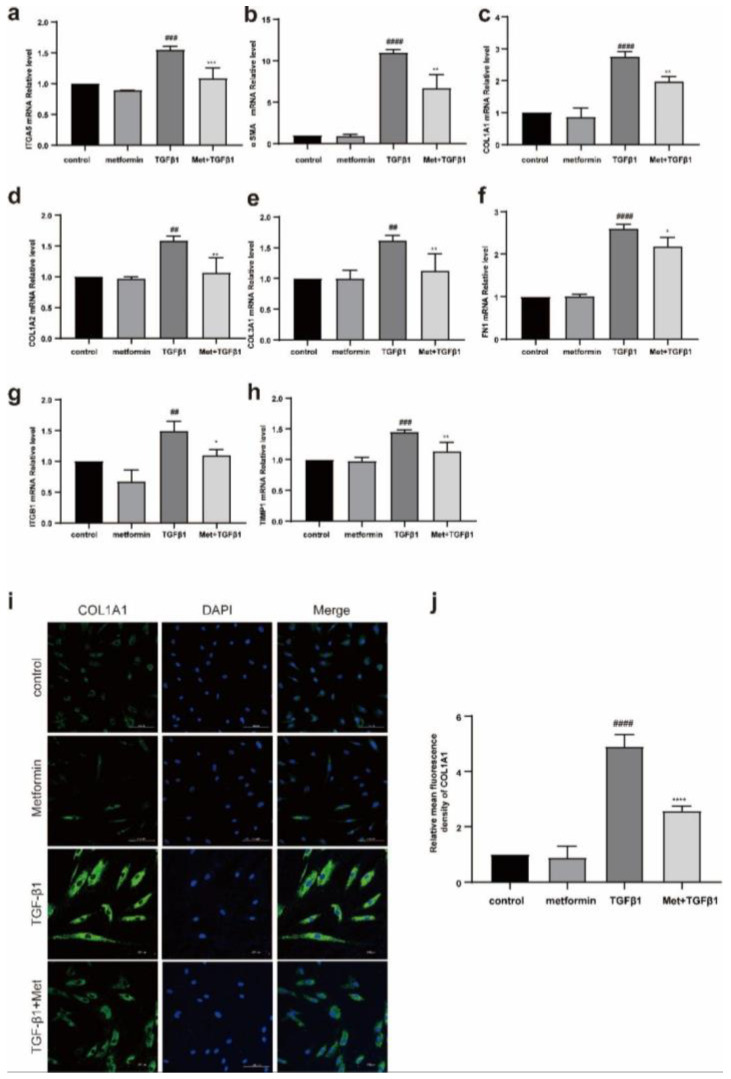
Metformin reduces the TGF-β-1-induced increases in extracellular matrix protein levels in fibroblasts. (**a**–**h**) TAO-OFs and NC-OFs were pretreated with metformin for 24 h and then stimulated with 5 ng/mL TGFβ1 for 48 h. A quantitative analysis of the mRNA levels of profibrotic cytokines. (**i**) IF analysis of the expression of COL1A1. Scale bar, 100 µm. (**j**) Quantitative analysis of the results shown in (**b**). *n* = 3 in each group. ## *p* < 0.01, ### *p* < 0.001, #### *p* ≤ 0.0001 compared with the control group, * *p* < 0.05, ** *p* < 0.01, *** *p* < 0.001, **** *p* ≤ 0.0001 compared to the TGF-β1 group, ns: not significant (ANOVA).

TAO OFs were stimulated with TGF-β1 (5 ng/mL). qPCR showed that metformin significantly inhibited the mRNA expression of TGF-β1-induced fibrosis-related molecules, including αSMA, ITGA5, COL1A1, COL2A1, COL3A1, FN1, and ITGB1 (Figure 5a–h). Moreover, metformin decreased the levels of fibrosis-related proteins, including COL1A1 (Figure 5i,j).

### 2.6. Metformin Activates Autophagy in Fibroblasts

**Figure 6:**
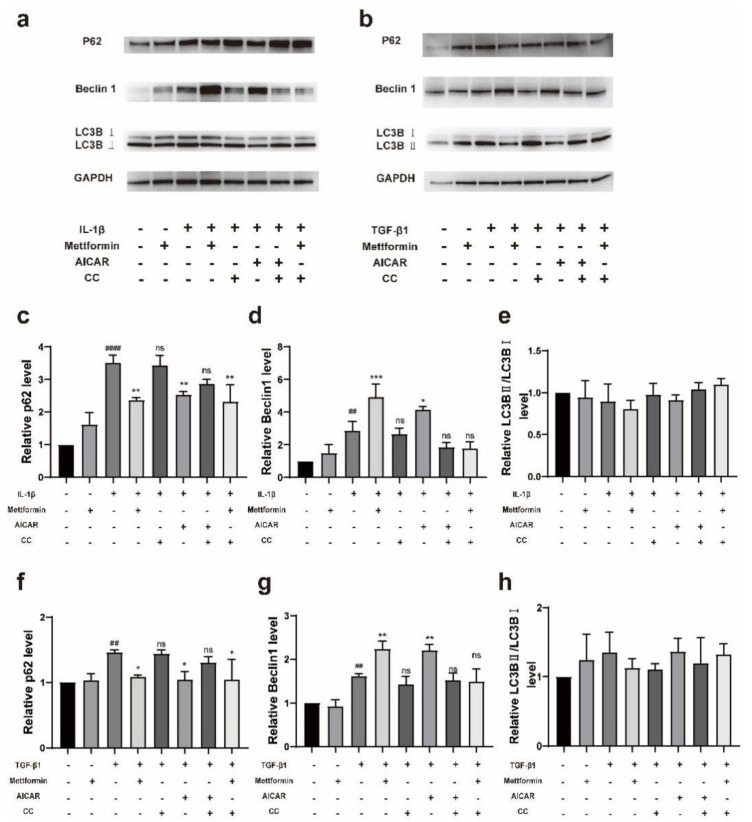
Metformin induces autophagy. (**a**) The protein levels of p62, Beclin1, and LC3B in fibroblasts pretreated with metformin, AICAR and CC before stimulation with 1 ng/mL IL-1β for 3 h were examined by Western blotting. (**b**) The same proteins in fibroblasts pretreated with metformin, AICAR, and CC before stimulation with TGF-β1 for 3 h were examined by Western blotting. (**c**–**e**) Quantitative analysis of the results shown in (**a**). (**f**–**h**) Quantitative analysis of the data shown in (**b**). The data are presented as the means ± SD. *n* = 3 in each group. ## *p* < 0.01, #### *p* ≤ 0.0001 compared with the control group, * *p* < 0.05, ** *p* < 0.01, *** *p* < 0.001, compared to the stimulated group, ns: not significant (ANOVA).

Western blotting showed that both metformin and AICAR significantly downregulated the level of p62, and a significant increase in Beclin-1 expression was found in both the inflammation model stimulated with IL-1β and the fibrosis model stimulated with TGF-β1 (Figure 6a–d,f,g). Moreover, IL-1β, TGF-β1, and CC partially restored the level of p62 and inhibited the increase in Beclin-1. However, the Lc3-II/Lc3-I ratio, which is another autophagy marker, was not significantly affected by these drugs (Figure 6e,h). Our findings suggest that metformin may activate autophagy and furthermore affect TAO inflammation and fibrosis.

### 2.7. Metformin Regulates the IL-1β-Mediated Inflammatory Response by Activating the AMPK/mTOR Signaling Pathway

**Figure 7:**
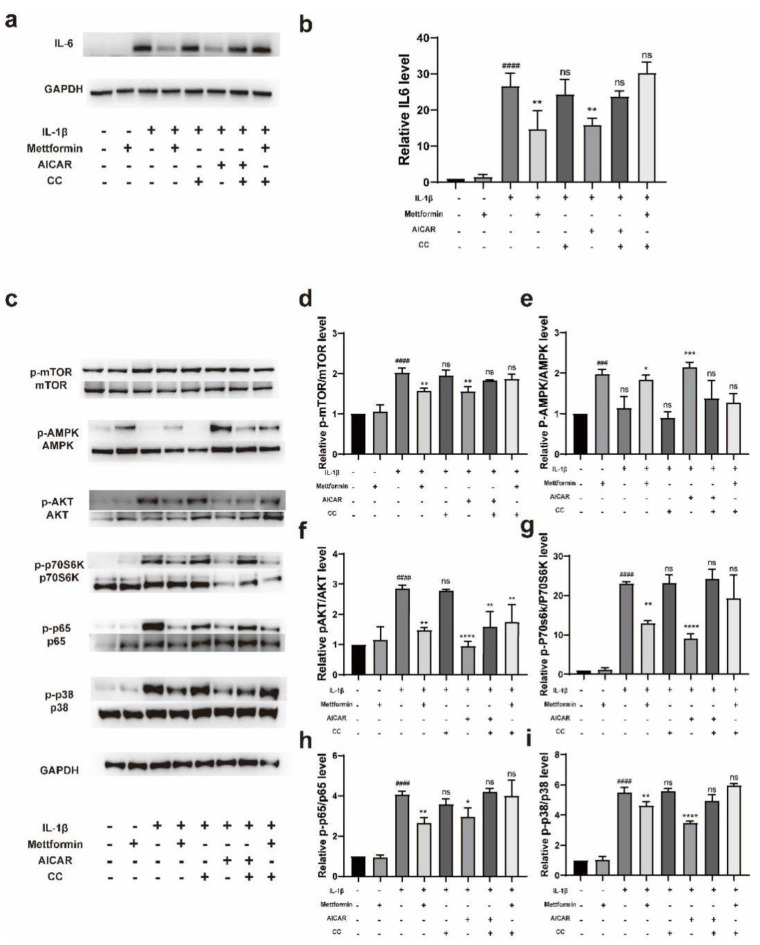
Metformin suppresses inflammation via the AMPK/mTOR signaling pathway. (**a**) TAO-OFs were pretreated with metformin for 24 h and then stimulated with 1 ng/mL of IL-1β for 24 h. The expression levels of inflammation-associated proteins were determined by Western blotting. (**b**) Statistical analysis of the results in (**a**). (**c**) The protein levels of AMPK, p-AMPK, mTOR, p-mTOR, p70S6K, p-p70S6K, p65, p-p65, p38, p-p38, AKT, and p-AKT in cells pretreated with metformin, AICAR, and CC before stimulation with IL-1β for 3 h were examined by Western blotting. (**d**–**i**) Quantification of the results in (**c**). The data are presented as the means ± SDs. *n* = 3 in each group. ### *p* < 0.001, #### *p* < 0.0001, compared with the control group, * *p* < 0.05, ** *p* < 0.01, *** *p* < 0.001, **** *p* < 0.0001 compared with the stimulated group, ns: not significant (ANOVA).

We hypothesized that metformin might downregulate TAO inflammation in an AMPK-dependent manner. To confirm the role of metformin in activating the AMPK signaling pathway in TAO OFs, we pretreated cells with AICAR (250 µM), metformin (1000 µM) or compound C (100 nM) for 24 h and then stimulated the cells with IL-1β (1 ng/mL) for 24 h. The Western blot results showed that IL-1β significantly stimulated the expression of IL-6, whereas the AMPK activator metformin and AICAR inhibited this inflammatory stimulation, and the AMPK inhibitor CC attenuated the ability of metformin and AICAR to downregulate the levels of inflammatory proteins (Figure 7a,b). To further determine the signaling pathway that depends on AMPK and the differentiation of myofibroblasts, IL-1β (1 ng/mL) was administered for 3 h after the same treatment as previously described. The results showed that metformin and AICAR activated AMPK and inhibited the phosphorylation of mTOR, and these effects further inhibited the downstream activation of p38, p65, p70S6K, and AKT. Conversely, IL-1β and CC inhibited the activation of AMPK (Figure 7c–i). Interestingly, we found that CC did not exacerbate the IL-1β-induced activation of these pathways. Moreover, our examination of p-AKT unexpectedly revealed that the preventive effect of metformin in cells was not attenuated by pretreatment with CC (Figure 7c,f).

### 2.8. Metformin Regulates TGF-β1-Mediated Fibrosis by Activating the AMPK/mTOR Signaling Pathway

**Figure 8:**
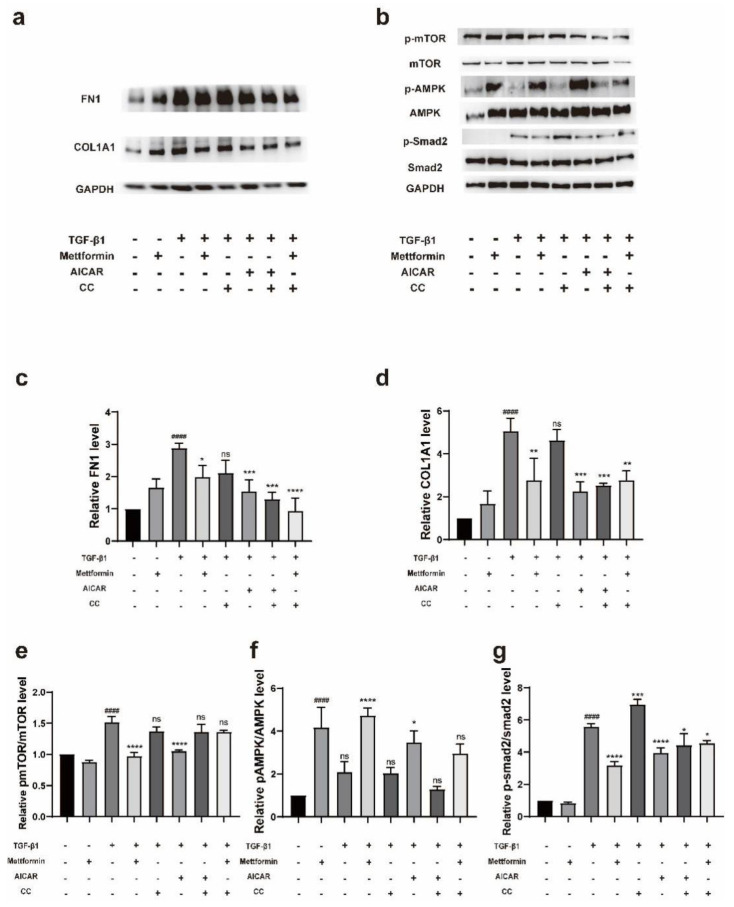
Metformin suppresses fibrosis via the AMPK/mTOR signaling pathway. (**a**) TAO-OFs were pretreated with metformin for 24 h and then stimulated with 5 ng/mL of TGFβ1 for 72 h. The expression level of fibrosis-associated proteins was determined by Western blotting. (**b**) The protein levels of AMPK, p-AMPK, mTOR, p-mTOR, Smad2, and p-Smad2 in cells pretreated with metformin, AICAR or CC before stimulation with TGF-β1 for 3 h were examined by Western blotting. (**c**) Statistical analysis of the results in (**a**). (**d**–**g**) Quantitative analysis of the results shown in (**b**). The data are presented as the means ± SDs. *n* = 3 in each group. #### *p* < 0.0001 compared with the control group, * *p* < 0.05, ** *p* < 0.01, *** *p* < 0.001, **** *p* < 0.0001 compared with the stimulated group, ns: not significant (ANOVA).

We pretreated cells with AICAR (250 µM), metformin (1000 µM) or compound C (100 nM) for 24 h and then stimulated the cells with TGF-β1 (5 ng/mL) for 72 h. Western blotting showed that the AMPK activators metformin and AICAR inhibited the expression of collagen type I and fibronectin in TGF-β1-stimulated fibroblasts, while the AMPK inhibitor CC did not attenuate the effects of metformin and AICAR (Figure 8a,c,d). To further determine the signaling pathway affected by AMPK, TGF-β1 (5 ng/mL) was administered for 3 h after the same treatment. The results showed that metformin and AICAR activated AMPK, inhibited the phosphorylation of mTOR, and further inhibited the activation of downstream Smad2. TGF-β1 and CC activated Smad2 (Figure 8b,e–g). Interestingly, we observed that metformin still decreased the TGF-β1-induced p-Smad2 levels when administered with CC (Figure 8b,g).

## 3. Discussion

In this study, we performed the first comparison of the activity of AMPK in the orbital connective tissue of TAO patients and controls and confirmed that the AMPK activator metformin exerted significant anti-inflammatory and antifibrotic effects on TAO in an in vitro OF model. In addition, the activation of autophagy by metformin also suggests a complex mechanism through which metformin regulates inflammation and fibrosis. To our knowledge, this study constitutes the first exploration of the relationship between AMPK activity and TAO and the multiple effects of the metformin-dependent AMPK/mTOR pathway on TAO.

Previous studies have suggested that AMPK may act as a key metabolic switch that promotes the resolution of established fibrosis by shifting the balance from anabolism to catabolism. We found differences in AMPK activity in the periorbital tissue of TAO patients by multicolor IF and IHC, which suggests that changes in AMPK activity are closely related to changes in pathological disease, and these findings are consistent with the results from a study of IPF [33]. Therefore, we speculate that increased AMPK activity can reduce inflammation and fibrosis in TAO.

IL-1β, which can be produced by macrophages, increases the secretion of inflammatory cytokines and induces the accumulation of glycosaminoglycans. Moreover, IL-1β constitutes one of the important pathogenic mechanisms of inflammation and edema in TAO orbital soft tissue. In this study, we found that metformin pretreatment significantly reduced the mRNA levels of cytokines stimulated by IL-1β and reduced inflammation at the protein level; these results are similar to previous findings in uveitis [37], other autoimmune diseases [38,40,42,43,44,45], and TAO [46]. Notably, TAO OFs stimulated with IL-1β showed more significant increases in most inflammatory indicators compared with NC-OFs. In addition, metformin exerted different effects on improving inflammation between the two groups, and the specific mechanism remains to be determined. Moreover, we surprisingly found that compound C did not reverse the inhibition of AKT phosphorylation induced by metformin, which suggests that metformin may first inhibit AKT to activate AMPK and thereby plays an anti-inflammatory role.

During the inflammatory progression of TAO, HA, which is a glycosaminoglycan, is involved in many pathological processes [47]. Studies have evaluated the histopathological changes in TAO, and the accumulation of HA in orbital connective tissue and extraocular muscles was observed, which aggravated retrobulbar edema in TAO patients [48]. We hypothesized that the activation of AMPK exerted a certain inhibitory effect on the production of extracellular matrix components, such as glycosaminoglycans, and verified this hypothesis at the protein and mRNA levels by ELISA and qPCR. This finding is consistent with previous research by Han et al. [46]. Han’s team also proved that metformin exerts an antiadipogenic in TAO in vitro via ERK inactivation [46].

TGF-β1 can be secreted by macrophages or T cells and plays a vital role in fibrotic tissue remodeling during the late stage of TAO. Some scholars have proposed that metformin prevents fibrosis by targeting the TGF-β1 signaling pathway [49]. In this study, we found that AMPK activity was decreased by the administration of metformin with CC, but this treatment reduced the level of TGF-β1-induced p-Smad2, which suggests that metformin can attenuate p-Smad2 independent of the AMPK pathway, and this finding is consistent with the abovementioned findings.

Autophagy plays a very important role in various physiological and pathological processes [50], and metformin reportedly prevents or treats tumors and metabolic diseases by activating autophagy [51,52,53,54,55,56]. Western blotting was performed to examine the responses of p62, Beclin1, and LC3-II/LC3I to AMPK activators (metformin and AICAR) and AMPK inhibitors (CC). Interestingly, we found that the level of Beclin1 also increased to a certain extent after IL-1β or TGF-β1 stimulation, and treatment with metformin or AICAR increased this change. We hypothesize that the level of autophagy stimulated by IL-1β or TGF-β1 is not sufficient to resist the effect of the disease and can even exacerbate pathological progression, but the further activation of autophagy by metformin can play a complex role in the treatment of inflammation and fibrosis.

Notably, the study conducted by Hong Li reported that some drugs, such as icariin, prevented the pathological progression of TAO by inhibiting AMPK to inhibit autophagy [57]. Autophagy may play dual roles in inflammatory diseases. The double effects of autophagy on TAO further reflect the complexity of the pathogenesis and provide new ideas for future research on the mechanism. In addition, it is worth noting that we examined intracellular LC3B protein expression at a single time point and did not observe a significant increase in the ratio of LC3-II to LC3-I after metformin pretreatment. We believe that LC3-II may have been degraded by lysosomes together with the encapsulated contents at the time point examined. Research on the dynamics of autophagic flow in TAO may require more in-depth examination.

Metformin is a first-line therapy for type 2 diabetes mellitus (T2DM), and the tight association between TAO and diabetes mellitus (DM) has been popular these days. Since 1999, it has been suggested that diabetes is an important risk factor in patients with TAO [58]. Both TAO and type 1 diabetes mellitus (T1DM) are autoimmune diseases, and they share some susceptibility and associated loci of the HLA system [59,60]. In addition, several studies reported patients that have both TAO and DM, showing a close relationship between them, and TAO is more frequent and severe in T2DM [61,62]. T2DM causes increased lipogenesis and inflammation, as well as insulin resistance, overweight, and compensatory hyperinsulinemia. These typical features are all closely related to TAO. Elevated insulin in DM reduces IGF-1 binding protein and increases IGF-1 bioavailability [63], followed by an increase in adipogenesis and the overproduction of the extracellular matrix, finally causing an increase in the orbital volume. It has been demonstrated in vitro that TSHR and IGF1R activation causes a series of effects through the phosphatidylinositol 3 kinase (PI3K) and mTOR pathways in human orbital fibroblasts, and inhibitors of these pathways reduce hyaluronan accumulation and adipogenesis [64]. Our study also confirmed that metformin could play a series of therapeutic roles in downregulating mTOR levels by activating AMPK. We suspected that metformin could serve as a nonimmunosuppressive treatment to reduce the severity of conditions in TAO patients with DM. The similar pathogenesis between TAO and T2DM also implied that metformin could play a therapeutic role in TAO patients through the same molecular mechanism.

Many clinical studies conducted to date have shown that metformin significantly reduces TSH levels without affecting the thyroxine levels in hypothyroid patients with diabetes mellitus but does not affect the TSH levels in the peripheral blood of euthyroid diabetic patients [65,66,67,68,69]. Additionally, the use of metformin does not worsen the condition of patients with hyperthyroidism and diabetes [70]. In addition, metformin treatment reportedly decreases the thyroid nodule volume, reduces the risk of thyroid nodules and exhibits antiproliferative activity [71,72,73]. Dunta et al. suggested that the effect of metformin on decreasing the TSH levels might be explained by the metformin-induced activation of AMPK [74].

The effects of metformin on thyroid-related diseases are remarkable; however, there is currently no effective evidence showing the independent effects of metformin in TAO patients with diabetes. We look forward to a follow-up retrospective analysis of TAO patients with diabetes, which will explore the effects of metformin on diseases, as well as prospective clinical studies on the use of metformin in TAO patients. More clinical trial evidence is still needed to unravel the mystery of the story.

Our study provides evidence showing that AMPK activation is deficient during progressive inflammation and fibrosis in TAO patients and that AMPK activators such as metformin can ameliorate the pathological damage of this disease. However, one limitation is that animal models do not perfectly mimic TAO, and more in-depth mechanism-specific studies, including an evaluation of the efficacy and safety using animal models, are still needed. In general, our experiments indicate that AMPK may become a promising therapeutic target for TAO, and our in vitro studies provide strong evidence of the mechanism and therapeutic effects of metformin on TAO.

## 4. Materials and Methods

### 4.1. Subjects and Enrolment Criteria

We obtained orbital connective tissues from patients with TAO (*n* = 15) and from controls (*n* = 10). The TAO patients underwent orbital decompression, and patients who underwent eyeball enucleation were used as controls. The baseline characteristics of the patients are summarized in Table 1.

All subjects signed the informed consent form before participation. This study was conducted in accordance with the Declaration of Helsinki and was approved by the Institutional Review Board of Zhongshan Ophthalmic Center (2016KYPJ028).

### 4.2. Cell Culture and Treatment

The cultured cells were used between the 3rd and 6th passages. DMEM with 20% FBS (Gibco, Waltham, MA, USA) was used when we started the cultures from frozen cell stocks or from tissues, and we changed to 10% FBS when we pretreated the cells with or without metformin (Sigma–Aldrich, St. Louis, MO, USA), 5-aminoimidazole-4-carboxamide 1-β-D-ribofuranoside (AICAR) or compound C (Selleck Chemicals, Houston, TX, USA) for 24 h to ensure normal growth. We changed to 1% FBS before the cells were stimulated with or without IL-1β (1 ng/mL) or TGF-β1 (5 ng/mL) (R&D systems, Minneapolis, MN, USA).

### 4.3. Cell Viability Assay

The cell viability was examined with a Cell Counting Kit-8 assay (CCK8, Beyotime Institute of Biotechnology, Shanghai, China) according to the manufacturer’s instructions. The cells were exposed to different concentrations (0, 25, 50, 100, 200, 400, 1000, 2000 µM) of metformin for different times (24, 48, 72 h). The percent viability was calculated using the following formula:

Cell viability (%) = A450 nm treated/A450 nm control × 100%

### 4.4. Immunofluorescence (IF) Analysis

The cells were inoculated on slides and grown to approximately 70% confluence. The slides were washed twice with phosphate-buffered saline (PBS) (Gibco, Waltham, MA, USA). The slides were fixed with 4% paraformaldehyde for 20 min, blocked with 3% BSA for 30 min, and incubated with primary antibodies against p-AMPK, collagen I, IL-8 (Cell Signaling Technology, Boston, MA, USA), vimentin, desmin, keratin, and S100 calcium binding solution (Thermo Fisher Scientific, Rockford, IL, USA) overnight at 4 °C. The slides were washed again and incubated with CY3-labeled goat anti-mouse secondary antibodies (Servicebio, Wuhan, China), goat anti-mouse IgG H&L (Alexa Fluor 647), and goat anti-rabbit IgG H&L (Alexa Fluor 488) (Thermo Fisher Scientific, Rockford, IL, USA) at room temperature in the dark for 1 h. Nuclei were stained for 5 min with DAPI (Servicebio, Wuhan, China). After a final wash, an anti-fluorescence quenching agent (Servicebio, Wuhan, China) was added to mount the slides, and images were collected with a microscope (Nikon, Tokyo, Japan).

### 4.5. Immunohistochemistry (IHC)

Paraffin-embedded tissues were cut into 3 µm thick sections and placed in xylene for dewaxing, absolute ethanol for rehydration, and EDTA (pH 9.0) antigen retrieval solution (Servicebio, Wuhan, China) for antigen retrieval. The sections were washed three times with PBS, placed in 3% H_2_O_2_, incubated at room temperature in the dark for 25 min, and blocked with 3% BSA at room temperature for 30 min. The slices were incubated with an antibody against p-AMPK overnight at 4 °C. The slices were washed three times and incubated with a goat anti-mouse IgG secondary antibody at room temperature for 1 h. DAB was then added, and color development was stopped after 45 s. Then, the cell nuclei were counterstained with hematoxylin, and the sections were dehydrated and sealed with neutral gum.

### 4.6. RNA Isolation and Quantitative Real-Time Polymerase Chain Reaction (qRT–PCR)

Total RNA was extracted using an RNA-Quick Purification Kit (ES Science, Shanghai, China) and then reverse transcribed into cDNA by using a reverse transcription kit (TaKaRa, Dalian, China). qRT–PCR was performed using TB Green Premix Ex Taq II (TaKaRa, Dalian, China). GAPDH was used as the housekeeping gene. The primer pairs used are listed in Table 2.

### 4.7. Cytokine Quantification by Enzyme-Linked Immunosorbent Assay (ELISA)

The concentrations of IL-6, IL-8, and HA in the cell culture supernatant were quantified using ELISA kits (R&D Systems, Minneapolis, MN, USA) according to the manufacturer’s instructions.

### 4.8. Western Blotting

A protein extraction kit (KeyGEN, Nanjing, China) was used to lyse the cells. Protein concentrations were measured with a BCA kit (Beyotime, Shanghai, China). The proteins were separated by SDS–PAGE and transferred to a PVDF membrane. The membrane was blocked with 5% skim milk for 1 h at room temperature and was then incubated with antibodies against AMPK, p-AMPK (Thr172), mTOR, p-mTOR (Ser2448), NF-κBp65, phospho-NF-κBp65 (Ser536), p38 MAPK, phospho-p38 MAPK (Thr180/Tyr182), p70S6 kinase, phospho-p70 S6 kinase (Thr389), AKT, phospho-AKT (Ser473), Smad2, phospho-Smad2 (Ser465/467), β-actin, Beclin-1, LC3B, SQSTM1/p62 (Cell Signaling Technology, Beverly, MA, USA), fibronectin, α-smooth muscle actin (α-SMA), GAPDH, and human collagen (type I) (Thermo Fisher Scientific, Rockford, IL, USA) at 4 °C overnight. The membrane was then incubated with a goat anti-rabbit secondary antibody or a horse anti-mouse secondary antibody (Cell Signaling Technology, Boston, MA, USA) at room temperature for 1 h. Finally, the signals were examined using a chemiluminescence kit (Bio–Rad, Hercules, CA, USA).

### 4.9. Statistical Analysis

Each experiment was performed in triplicate, and the data are expressed as the means ± SD. GraphPad Prism software was used to analyze the results. Statistical analysis was performed with one-way ANOVA and an unpaired t test. A *p* value ≤ 0.05 was considered to indicate a statistically significant difference.

## Figures and Tables

**Table 1:** Features of patients with TAO and normal controls undergoing surgery.

	TAO	NC	*p*
No. of participants	15	10	
Sex, M/F	9/6	3/7	0.2262
Age, mean ± SD, (range), y	47.47 ± 7.846 (34–61)	43.00 ± 13.44 (28–70)	0.3120
Clinical activity score			NA
Stable	8		
Active	7		
Duration of TAO, median (range), m	28 (2–98)		NA
Therapy history			NA
Thyroid surgery	1/15		
Orbital irradiation	2/15		
Steroid treatment	5/15		
Thyroid function			NA
Hyperthyroid	1/15		
Hypothyroid	0/15		
Euthyroid	14/15		

TAO, Thyroid-associated ophthalmopathy; NC, normal control; M, male; F, female; SD, standard deviation; y, year; m, month; NA, not applicable.

**Table 2:** Primer Sequences of RT-qPCR.

Genes	Sequences (5′-3′)
IL-6	F: CACTGGTCTTTTGGAGTTTGAG R: GGACTTTTGTACTCATCTGCAC
IL-8	F: CCACCGGAAGGAACCATCTC R: GGGGTGGAAAGGTTTGGAGT
CCL2	F: CCTTCATTCCCCAAGGGCTC R: CTTCTTTGGGACACTTGCTGC
CXCL1	F: TTCACAGTGTGTGGTCAACAT R: AAGCCCCTTTGTTCTAAGCCA
CXCL2	F: AGTGTGTGGTCAACATTTCTCA R: GCTCTAACACAGAGGGAAACAC
HAS1	F: GCGGGCTTGTCAGAGCTAC R: ACTGCTGCAAGAGGTTATTCC
HAS2	F: CCTCCTGGGTGGTGTGATTT R: GCGTCAAAAGCATGACCCAA
ITGA5	F: GGCTTCAACTTAGACGCGGAG R: TGGCTGGTATTAGCCTTGGGT
α-SMA	F: GGGACTAAGACGGGAATCCT R: TGTCCCATTCCCACCATCAC
COL1A1	F: AAAGATGGACTCAACGGTCTC R: CATCGTGAGCCTTCTCTTGAG
COL1A2	F: CTCCATGGTGAGTTTGGTCTC R: CTTCCAATAGGACCAGTAGGAC
COL3A1	F: CGCCCTCCTAATGGTCAAGG R: TTCTGAGGACCAGTAGGGCA
FN1	F: ACAAGCATGTCTCTCTGCCAA R: GCAATGTGCAGCCCTCATTT
ITGB1	F: CTGTGATGCCTTACATTAGCAC R: ATCCAAATTTCCAGATATGCGC
TIMP1	F: CATCACTACCTGCAGTTTTGTG R: TGGATAAACAGGGAAACACTGT
GAPDH	F: TTGCCATCAATGACCCCTT R: CGCCCCACTTGATTTTGGA

F, forward; R, reverse.

## Data Availability

The datasets generated during and/or analyzed during the current study are not publicly available but are available from the corresponding author on reasonable request.
